# Factors Associated with Suicide Attempts in Adults with ADHD: Findings from a Clinical Study

**DOI:** 10.3390/medicina61071178

**Published:** 2025-06-29

**Authors:** Camilla Perotti, Gianluca Rosso, Camilla Garrone, Valerio Ricci, Giuseppe Maina, Gabriele Di Salvo

**Affiliations:** 1 Department of Neurosciences “Rita Levi Montalcini”, University of Turin, 10126 Turin, Italy; camilla.perotti@unito.it (C.P.); gianluca.rosso@unito.it (G.R.); camilla.garrone@unito.it (C.G.); valerio.ricci@unito.it (V.R.); giuseppe.maina@unito.it (G.M.); 2 Psychiatric Unit, San Luigi Gonzaga University Hospital, Orbassano, 10043 Turin, Italy

**Keywords:** Attention-Deficit/Hyperactivity Disorder (ADHD), suicide attempt (SA), attempt methods, personality disorder comorbidity, psychiatric hospitalization, sociodemographic determinants

## Abstract

*Background and Objectives:* Suicidality in adults with Attention-Deficit/Hyperactivity Disorder (ADHD) is an emerging clinical concern, yet its mechanisms and risk factors are not fully understood. Specifically, little is known about the characteristics of suicide attempts (SAs), including the use of violent/nonviolent methods. This study aimed to investigate the prevalence and methods of SA in adults with ADHD and to identify associated sociodemographic and clinical factors. *Materials and Methods:* The sample included 211 adult outpatients with ADHD. Patients were grouped based on the presence/absence of a lifetime SA. Among attempters, those who used a violent method (e.g., hanging, shooting, or jumping from a height) were compared with those who used a nonviolent method (e.g., poisoning). Statistical analyses included χ^2^ tests, Kruskal–Wallis tests, and logistic regression. *Results:* In total, 9.9% (n = 21; 95% CI: 4.5–10.4) of participants reported a lifetime SA, with 23.8% (n = 5; 95% CI: 4.8–41.9) using violent methods. SA was significantly associated with comorbid personality disorders (*p* = 0.006, OR: 6.613, 95% CI: 0.537–5.812) and a higher number of hospitalizations (*p* = 0.008, OR: 1.980, 95% CI: 0.296–2.675). Nonviolent methods were linked to low self-esteem (*p* = 0.008). No significant associations with ADHD features or other psychiatric comorbidities emerged. *Conclusions:* Adults with ADHD are at risk for SA, showing patterns similar to other psychiatric populations. Unlike suicidal ideation, which has been directly linked to ADHD in previous studies, the transition to an SA appeared to be associated with comorbid personality disorders.

## 1. Introduction

Attention-Deficit/Hyperactivity Disorder (ADHD) is a neurodevelopmental condition that often persists into adulthood [1], significantly impacting various aspects of life, including emotional regulation, interpersonal relationships, and occupational functioning. ADHD has an estimated prevalence of 4% to 7% in childhood and 2.5% in adulthood, based on large-scale epidemiological studies [2,3]. ADHD is categorized into three subtypes: predominantly inattentive, predominantly hyperactive–impulsive, and combined presentations [4]. While it is primarily recognized for its core symptoms of inattention, hyperactivity, and impulsivity [5], research has increasingly highlighted its association with comorbid psychiatric disorders such as depression, anxiety, and substance use disorders [6].

These comorbidities, along with the core characteristics of ADHD, contribute to the increasing evidence linking ADHD to suicidal ideation (SI) and suicidal behaviors (SBs), which include actual suicide attempts (SAs), interrupted attempts, aborted attempts, completed suicides, preparatory behaviors, and non-suicidal self-injury behaviors. However, the nature of the relationship between ADHD and suicidality is not yet fully understood.

Unlike other psychiatric disorders, adult ADHD treatment varies widely across countries, mainly due to medication availability, especially amphetamines. In Italy, amphetamines are not marketed, and until about 18 months ago, atomoxetine was the only approved adult ADHD medication. Methylphenidate was prescribed off-label or to those who began treatment before 18. Only recently, methylphenidate was approved for adults starting treatment after 18. Besides limited medication options, a critical issue in ADHD treatment is the challenging transition from child to adult psychiatric services, which are often scarce and hard to access. This gap can cause treatment interruption, increasing SA risk due to the vulnerability of this developmental stage and loss of familiar clinical support.

Population- and community-based studies have consistently shown that adults with ADHD are at a higher risk of attempting suicide [7,8,9,10,11]. However, data on SB within clinical settings remain limited.

A recent meta-analysis highlighted a lifetime risk of SB in ADHD patients, both adults and children, of 18.9—95% CI: 14.2–24.6 (significantly higher compared to non-ADHD individuals, who had a rate of 9.3—95% CI: 5.2–16.2) [12]. Nonetheless, the literature on SB in adults with ADHD is highly heterogeneous: reported lifetime rates of SB among adults with ADHD range from 9.1% [13] to 51.5% [14].

Furthermore, despite growing interest in this phenomenon, it remains unclear whether the relationship between ADHD and suicidality (both SI and SB) is direct or mediated by psychiatric comorbidities. While some studies have suggested that this association is largely influenced by psychiatric comorbidities [10,15,16,17,18], other research has failed to support them, suggesting a direct association between the two phenomena [7,9,19]. Finally, other studies have hypothesized that psychiatric comorbidities are confounding but not responsible for the ADHD–suicidality relationship [12,18,19,20,21].

More specifically, after considering psychiatric comorbidities and psychosocial adversity, Ziegler et al. (2024) [15] agree that ADHD symptoms, such as inattention and impulsivity, are not directly linked to SB. Instead, they suggest that SB is influenced more by psychological and psychosocial factors and psychiatric comorbidities (with neuroticism as a risk factor). Other studies concluded that the link between ADHD and suicidality is mediated by the cumulative effect of externalizing disorders, such as conduct and oppositional defiant disorders [22], substance use disorders, affective disorders (emotional problems in males and depression in females) [23], or somatic conditions [24], rather than being accounted for by ADHD per se.

This evidence suggest that SB in ADHD may not be directly related to ADHD-specific traits such as impulsivity, inattention, or motor control issues. Instead, it may stem from the broader challenges of living with ADHD, including its negative impact on psychosocial functioning and the heightened risk of developing comorbid psychiatric disorders.

Accordingly, our research group recently investigated suicidality in adult patients with ADHD [25], examining SI and SB using the Columbia Suicide Severity Rating Scale (C-SSRS). The findings revealed that the severity of inattentive symptoms (both in childhood and adulthood) was linked to lifetime SI, while hyperactivity and impulsivity did not show a direct association, nor did any psychiatric comorbidity. In this study we did not find any clinical or socio-demographic factors significantly associated with SB in adult patients with ADHD. However, it should be noted that the sample size for SB was small, and statistical power was limited, whereas the sample size for SI was larger.

Notably, recent studies indicate that the elevated risk of suicide attempts in ADHD patients often precedes the initiation of methylphenidate treatment and tends to decline during continued therapy, suggesting that suicidality is more closely linked to worsening psychiatric symptoms leading to treatment rather than the medication itself, which appeared to be protective [26].

Given the significant role that impulsivity and emotional dysregulation may play in SA, it is worth investigating whether adult patients with a hyperactive/impulsive component might be at higher risk of SA compared to those with predominantly inattentive symptoms. However, few studies in the literature have investigated the potential role of ADHD subtype in suicidality, with heterogeneous results. In particular, two studies have reported higher rates of SA in adolescents and young adults with the combined ADHD subtype compared to those with predominantly inattentive ADHD [27,28]. A study on adult patients, on the other hand, found a correlation with the combined ADHD subtype only for parasuicidal gestures and not for SA [15].

Regarding suicide methods, a substantial body of literature has explored various socio-demographic, clinical, environmental, genetic, and neurobiological factors linked to the use of violent suicide methods in individuals with psychiatric disorders [29]. It is well-documented that men are more likely than women to employ violent methods in both suicides and SA [30,31]. Additionally, violent suicides have been associated with higher levels of impulsivity, aggression, and substance abuse disorder [32,33,34,35].

Considering that impulsivity and emotional dysregulation are core or frequent features in patients with ADHD—who are often young males—understanding whether they are at higher risk for violent SAs, which are typically more lethal, is clinically important for risk assessment and prevention.

However, there is a lack of studies addressing risk factors for violent or nonviolent suicide, particularly in relation to ADHD, and it is not known whether ADHD-related SAs are more violent or impulsive. One single study highlighted no difference in medical lethality or method between groups of attempters with and without ADHD, although it did not explore the correlates of violent versus nonviolent SA [17].

Therefore, the prevalence of SB in ADHD and related factors remains unclear, as it remains controversial whether this association is direct or mediated by psychiatric comorbidities. Moreover, it is not known whether adult patients with ADHD tend to engage in more violent SAs, nor what the potential risk factors for this type of attempts might be.

Our study aims to fill this gap through the following objectives:

(1) To evaluate the prevalence and modes of SA in a sample of 211 adult patients with ADHD (primary aim);
(2) To identify sociodemographic and clinical characteristics associated with an increased risk of SA or with different modes in these patients (secondary aim).

While most of the studies focused on SB in general, we decided to specifically focus on SAs and their methods and did not consider parasuicidal gestures, preparatory behaviors, or completed suicides.

This study is especially pertinent to clinical practice, as our cohort was directly recruited from help-seeking outpatients with a broad spectrum of psychiatric comorbidities, providing valuable insights into the complexities of suicidality in this population.

## 2. Materials and Methods

### 2.1. Study Design and Sample

This was a cross-sectional observational study on 211 adult (≥18 years of age) outpatients with a diagnosis of ADHD according to the DSM-5-TR criteria [4]. The patients were enrolled at the regional reference center for ADHD in the Psychiatry Unit of San Luigi Gonzaga University Hospital, Orbassano (Turin), from July 2021 to December 2024.

The aims and procedures were explained to all the enrolled patients. Exclusion criteria comprised age, refusal to participate, and intellectual disability (IQ below 80).

The study protocol was approved by the local Ethical Committees (number 196/2024).

### 2.2. Assessment

Data were obtained through a semi-structured interview, which format covered the following areas:

(1) Sociodemographic data: age, sex, marital status, education level, and occupational status.
(2) Clinical features of ADHD: ADHD subtype; severity of symptoms in childhood and in adulthood (according to the “Diagnostic Interview for ADHD in Adults”—DIVA—which is a validated semi-structured interview for assessing current and childhood ADHD symptoms in adults, demonstrating good test–retest reliability (ICC = 0.85–0.90) and strong convergent validity with other ADHD diagnostic tools [36]); current occurrence of symptoms (measured through the ADHD rating scale IV—ADHD-RS IV—which is a self-report questionnaire widely used to assess the severity of ADHD symptoms, demonstrating excellent internal consistency (Cronbach’s α = 0.94) and good convergent validity with other ADHD measures [37]); impulsivity (measured by the Barratt Impulsiveness Scale-11—BIS-11 (α = 0.83)—a validated self-report questionnaire assessing cognitive, motor, and non-planning impulsiveness) [38]; ADHD-related symptoms such as mood swings, anger outbursts, low self-esteem (which was evaluated clinically and according to the specific section of Criterion C of DIVA), low tolerance of frustrations, and sleep onset insomnia; areas of functional impairment; age at ADHD diagnosis; age at first ADHD treatment; family history of psychiatric disorders.
(3) Psychiatric comorbidities: psychiatric comorbidities were determined according to the Italian version of the Structured Clinical Interview for DSM-5 Axis I Disorders (SCID-5) [39]. Personality status was assessed using the Millon Clinical Multiaxial Inventory (MCMI-III) [40].
(4) A history of SA, defined as self-destructive behavior with the intent to end one’s life, regardless of the resulting harm [41], was retrospectively evaluated for each patient, with a focus on the method of SB. Following Stenbacka et al. (2015) [42], SA methods were categorized as violent (hanging, shooting, jumping from a height or moving train, cutting, and drowning) or nonviolent (poisoning). For individuals who had made multiple attempts, the classification was based on the most violent attempt.

**Figure 1:**
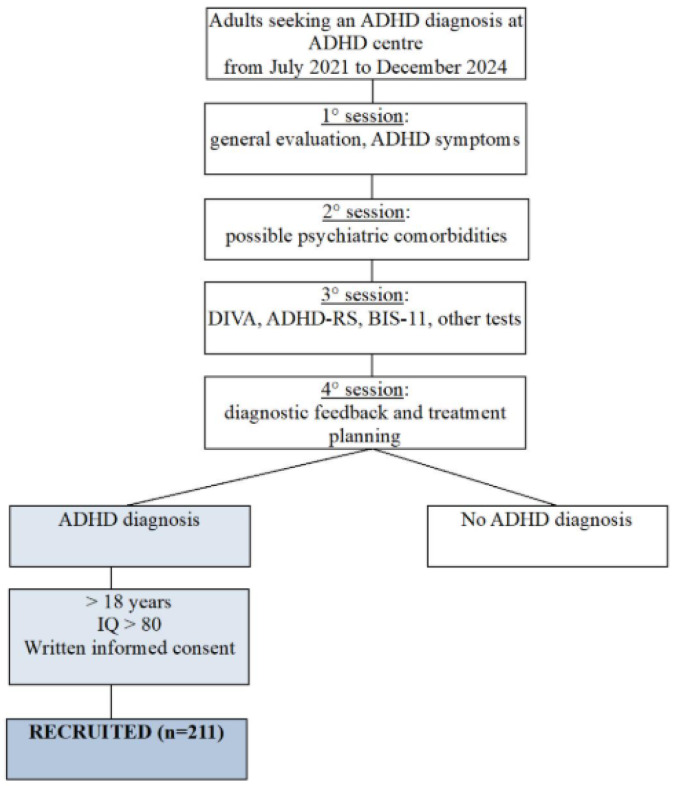
Recruitment process of the sample (n = 211).

The semi-structured interview, conducted by psychiatrists specializing in ADHD, is divided generally into four sessions (three to five depending on case complexity). The first session explores ADHD symptomatology in depth; the second focuses on possible psychiatric comorbidities; the third includes the administration of the DIVA (which was conducted—except for rare exceptions—in the presence of a caregiver who knew the patient in childhood), ADHD-RS, and other relevant tests, in addition to reviewing the elementary and middle school report cards; and the fourth session is dedicated to diagnostic feedback and treatment planning. The recruitment process is described in Figure 1.

### 2.3. Statistical Analysis

The sociodemographic and clinical features of the patients were summarized as means and SDs for continuous variables and as frequencies and percentages for categorical variables. We tested the distribution of continuous variables with the Kolmogorov–Smirnov test.

Patients were categorized based on whether they had a lifetime history of SA or had never attempted suicide. Additionally, patients who had used a violent suicide method were compared with those who had attempted suicide using a nonviolent method. Because the distribution was not normal (*p* < 0.001), comparisons were performed using χ^2^ tests for categorical variables and Kruskal–Wallis tests for continuous variables.

Binary logistic regression (with the Hosmer–Lemeshow goodness-of-fit test) was used to identify explanatory variables associated with lifetime history of SA. Significant variables were selected using a forward stepwise procedure. A probability of 0.05 was required for inclusion in the equation. The group comparison results were presented as two-sided *p*-values rounded to three decimal places. The criterion for statistical significance in all comparisons was a *p*-value < 0.05.

All statistical analyses were performed using SPSS software version 29.0.1.0.

## 3. Results

A total of 211 adult patients with a diagnosis of ADHD were enrolled in the study. The sample’s demographic and clinical features are shown in Table 1.

The lifetime prevalence of SA was 9.9% (n = 21, 95% CI: 6.2–13.8). Among suicide attempters, 23.8% (n = 5, 95% CI: 9.5–42.9) attempted suicide with a violent method.

The 72% (n = 30) of our sample showed comorbidity with at least one psychiatric disorder. The most common comorbidity was substance use disorder (30.3%), followed by major depressive disorder (27.5%) and personality disorders (14.2%). Among personality disorders, the most represented cluster was Cluster B, often with multiple traits. Specifically, 63.4% (n = 19) had a Cluster B Personality Disorder (11 borderline, 4 histrionic, and 4 antisocial), 3.3% (n = 1) had a Cluster A Personality Disorder, 3.3% (n = 1) had a Cluster C Personality Disorder, and 30% (n = 9) were diagnosed with Unspecified Personality Disorder. Among these “unspecified” patients, most showed predominant features of Cluster B.

Table 1 and Table 2 show the demographic and clinical features of the subgroups (ADHD with SA vs. ADHD without SA, ADHD with violent SA vs. ADHD with nonviolent SA), compared to χ^2^ tests or Kruskal–Wallis H tests. The variables with a statistically significant difference were subjected to binary logistic regression. When a single variable was significantly related to the method of SA, binary logistic regression was not performed for this subgroup.

The results of the binary logistic regression model are described in Table 3.

The incidence of SA appeared to be related to comorbidity with personality disorders (*p* = 0.006, OR: 6.613, 95% CI: 0.537–5.812) and the number of psychiatric hospitalizations (*p* = 0.008, OR: 1.980, 95% CI: 0.296–2.675).

The incidence of SA using nonviolent methods appeared to be related to low self-esteem (*p* = 0.008). Specifically, low self-esteem was reported by all patients with nonviolent SA (n =21, 100%) compared to three out of five patients with violent SA (60%).

As an indicator of effect size, we report the Nagelkerke R^2^. The Nagelkerke R^2^ value for logistic regression (patients with SA vs. patients without SA) was 0.478, which, according to Cohen’s guidelines, indicates a very large effect size. Furthermore, our power analysis for the logistic regression indicated a very high statistical power (92%).

No socio-demographic features, clinical features of ADHD, or other psychiatric comorbidities were significantly associated with the occurrence of SA or the method used. However, while the comparison between SA and non-SA groups demonstrates good statistical power and effect size, analyses involving the modes of SA have limited statistical power due to the small sample size and should therefore be interpreted with caution.

## 4. Discussion

In our sample, 9.9% of patients has a history of one or more SAs. This figure is consistent with our previous study [25], which reported a prevalence of 9.5%. However, it is lower than the average found in the meta-analysis, which indicated a prevalence of SB at 18.9% [12]. This difference might be due to the inclusion of studies on children and underage individuals (which is known to be a critical period for suicide risk, as it is the second leading cause of death for adolescents 15 to 19 years old [43]) and to the inclusion not only of SA but also parasuicidal gestures or non-suicidal self-injurious behaviors.

The SA prevalence in our ADHD sample is lower compared to what has been observed in other psychiatric disorders, such as bipolar disorder (33.9%) [44], major depressive disorder (31%) [45], and schizophrenia (26.8%) [46]. Therefore, despite the various biases to consider (such as the average age of the samples), adult ADHD patients appear to have a lower suicide risk than other psychiatric patients, even though they exhibit high levels of impulsivity and emotional dysregulation.

In our sample, SA did not appear to be significantly influenced—after logistic regression analysis—by ADHD subtype (although the combined subtype showed a higher rate of SA), symptom severity, related symptoms, or age at diagnosis. This is consistent with our previous study, which found no correlated factors [25]. This also supports Ziegler’s findings, which indicate that the core ADHD symptoms of inattention and hyperactivity–impulsivity were not associated with past SB [15].

Unlike SI, which appears to be linked to various severity aspects of ADHD [11,20,22,25,40,47], the occurrence of SA seems to be less related to ADHD itself. Instead, it appears to be more strongly associated with comorbidity and personality disorders, which is notably high in this population [48]. In this regard, it is not surprising that Cluster B was the most represented personality disorder cluster in our sample. Thus, while SI seems to stem directly from ADHD, the transition to actual SA appeared to be more influenced by personality disorder comorbidity and not by ADHD itself. It is interesting that the only comorbidity related with SA in adult ADHD patients is personality disorders, rather than depressive disorders or substance use disorders. This aligns with the findings of other studies, which have shown that individuals with SB and ADHD, compared to those without ADHD, are less likely to be diagnosed with depression and more likely to receive a clinical diagnosis of a personality disorder [17]. However, contrary to these evidence, other studies highlighted a significant association between SA, depression, and substance use disorder [7,10,49]. This difference may stem from the way the two disorders were categorized: while our study relied on clinical diagnoses (which are likely more restrictive), other studies based their findings on online questionnaires, with self-reported data on conditions like ADHD, depression, and anxiety, rather than clinical evaluations. Furthermore, other studies considered not only SA (as we did) but also parasuicidal gestures or non-suicidal self-injurious behaviors. Another possible interpretation of these differences takes into account the potential mediation of comorbidity with personality disorders in the relationship between SB and depression/substance use in ADHD patients. In this regard, another study found that the personality trait of neuroticism (the tendency to experience negative emotions in response to stress) in ADHD patients fully mediated the relationship between depression, substance use disorders, and SB [15], confirming that it is primarily the personality structure that determines the risk of suicide in these patients, rather than depression or substance use. This suggests that neuroticism may not only reflect a transdiagnostic vulnerability but may also interact with core ADHD symptoms by reducing stress resilience and increasing susceptibility to suicidal risk.

Regarding substance use, it is important to highlight that in our sample, while a substantial portion of patients use alcohol, sometimes inappropriately (21.3%), only a small percentage meet the criteria for an alcohol use disorder (5.2%), which is known to be one of the major risk factors for suicidality [50]. This is quite consistent with what is reported in the literature, which states that alcohol is not one of the most commonly used substances, even though its use is still significantly more frequent in ADHD patients compared to the general population [51]. Instead, the majority of ADHD patients use stimulants and cannabinoids, primarily for self-medication purposes. In fact, ADHD patients often use stimulants paradoxically to reduce and combat mental and physical restlessness, inattentiveness, and emotional moodiness [52], and they often use cannabinoids to reduce anxiety and restlessness or to sleep [51]. In support of this, a study highlighted a higher risk of SA in ADHD patients with alcohol dependence but not with dependence on other substances [19]. Therefore, considering the low prevalence of alcohol use disorder, the substances used, and their potential impact on symptomatology, it is not surprising that the presence of a substance use disorder did not emerge as a specific risk factor for SA in our sample.

Besides the comorbidity with personality disorders, the only other factor found to be correlated with SA in our analysis is the number of psychiatric hospitalizations, which was reasonably higher in patients with a history of SA, as it represents one of the most frequent indications for psychiatric hospitalization. This finding may also reflect a bidirectional relationship: while SAs often lead to psychiatric admission, repeated psychiatric hospitalizations may indicate more severe or treatment-resistant psychopathology, which are factors that are themselves associated with elevated suicide risk.

In our sample, among suicide attempters, 23.8% of patients attempted suicide with a violent method. For comparison, in a study on bipolar patients the percentage was 30.6% [53]. More generally, in a study on suicide attempters with various psychiatric diagnoses, almost one in every three attempters had made a violent SA in their lifetime [54]. Therefore, despite higher levels of emotional dysregulation and impulsivity, patients with ADHD in our sample did not exhibit a higher frequency of SAs using violent methods compared to other psychiatric patients. This is in line with the little evidence available, which shows similar SA methods in psychiatric patients with and without ADHD [17].

In our study, the only factor significantly associated with the method of SA was self-esteem: ADHD patients with low self-esteem were more likely to attempt suicide using nonviolent methods compared to those without low self-esteem. Although male gender is notably associated with more violent SA in the literature [30,31], it was not significantly related to an increased risk of such attempts in our sample. However, even though this relationship was not statistically significant, male patients did show a higher frequency of violent SA than females (30.8% vs. 12.5%). Given that low self-esteem was significantly more common in our female sample than in males, this may help explain why low self-esteem was associated with nonviolent SA. Another possible interpretation of this finding could be that patients with low self-esteem are less likely to feel confident in making permanent decisions, being too uncertain or anxious about the permanent consequences of a violent SA. This association may also reflect an internalizing profile, characterized by self-critical affect, poor emotion regulation, and rumination. According to Beck’s Cognitive Theory [55], negative self-schemas contribute to hopelessness and planned, non-impulsive suicidal behavior, consistent with our findings.

Given the small sample size, the statistical comparison between violent and nonviolent SAs should be interpreted with caution. For this reason, we deemed it appropriate to report some descriptive statistics comparing patients with violent versus nonviolent SA—beyond sex, already discussed—even though no statistically significant differences were found. As shown in Table 2, patients with violent SA started ADHD-specific treatment approximately five years later than the others, despite having a similar age at diagnosis. This finding raises questions about the potential protective role of methylphenidate in reducing SA risk, a topic already discussed and supported in the literature [26]. Moreover, patients with a history of violent SA had higher scores on the BIS-11, which measures impulsivity. This may suggest that impulsivity is not necessarily associated with SA risk per se but might play a more specific role in the risk for violent SA. This finding may also reflect the fact that our study assessed trait impulsivity, which might capture different aspects of impulsive behavior compared to task-based or situational measures typically used in the ADHD literature. Finally, a positive psychiatric family history appeared more frequent among patients with violent SA compared to those with nonviolent SA (100% vs. 75%, respectively). This may suggest a stronger genetic or familial vulnerability related to more violent SA, as highlighted by previous genetic studies [56,57].

Our results underscore the importance of conducting a comprehensive, 360-degree assessment of ADHD patients, regardless of the severity of their ADHD symptoms. For those with personality disorder, treatment should not only include pharmacological interventions or cognitive–behavioral psychotherapy but also incorporate targeted therapies for personality disorders, such as dialectical behavior therapy. Particular attention must be paid to ADHD patients with histories of psychiatric hospitalization, even when these are not directly related to suicide attempts. Furthermore, suicide risk should not be limited to those with impulsivity, substance use, or depression comorbidities; rather, clinicians should address not only core ADHD symptoms but also associated factors like self-esteem, which warrants thorough evaluation in this population.

Our study has several strengths, including a well-characterized clinical sample, drawn from a specialized center with expertise in adult ADHD, which reflects real-world psychiatric practice, enhancing the applicability of our findings to everyday clinical settings. Patients were assessed by experienced psychiatrists through a comprehensive clinical and diagnostic evaluation. This assessment covered not only ADHD but also possible psychiatric comorbidities. Diagnoses were made clinically, with the support of structured and validated tools such as the DIVA. In our study, we performed not only chi-square tests but also logistic regression analyses, with high statistical power, which provided a more in-depth understanding by estimating the strength and direction of these associations, also controlling for potential confounding variables, strengthening the consistency and interpretability of our findings. Furthermore, our results showed a very large effect size, which represents one of the key strengths of our study, demonstrating both strong explanatory power and robustness. However, our study should be considered in light of some limitations. First, the cross-sectional design does not allow causal relationships to be inferred or etiological factors to be assessed. Therefore, future longitudinal studies should address the relationship between ADHD, personality disorders, and suicide risk. It should be noted that, even if MCMI-III considers subthreshold personality traits, we have not considered them in our study. This could represent a limitation, as they could nonetheless influence the risk of suicide attempts. Another limitation is represented by the exclusion of completed suicides, which limits the generalizability of our findings to all suicidal outcomes. Moreover, we did not conduct a psychometric evaluation of the tests in our sample, relying instead on their established psychometric properties in the literature. Furthermore, in our analysis the impact of pharmacological treatment on SA was not evaluated, due to the very low proportion of patients who were receiving ADHD medication at the time of assessment. Evaluating the impact of treatment initiation on suicidality in our sample would be valuable in the future, for example, through collecting pre-study medication data. Limitations of the analysis of SA vs. non-SA are represented by the imbalance in group sizes (which may have affected the robustness of the statistical comparisons) and by the presence of some unstable variables in our model. However, the results remained consistent after rerunning the regression and excluding these variables. Regarding the comparison between violent and nonviolent SA, the small number of cases represents a major limitation, and future studies with larger samples are needed to clarify potential differences in suicide attempt methods. Finally, the retrospective assessment of suicide attempts may be subject to recall bias. In addition, cultural factors—such as the stigma associated with suicide in Italy and Europe more broadly [58]—may have contributed to underreporting. A similar recall bias may apply to the reconstruction of childhood ADHD symptoms; however, the involvement of caregivers and access to school records helped minimize this issue.

In the context of ADHD, it is important to highlight that, more than in other disorders, substantial differences exist between countries—particularly concerning the availability of medications, as well as access to healthcare services and waiting times. These disparities may limit the comparability of findings across nations, constituting a significant methodological limitation. As our study did not evaluate the impact of treatment on suicide risk, and since age at diagnosis or treatment initiation did not influence suicide risk in our sample, we consider that our results may nonetheless be generalizable to populations with adequate access to care.

## 5. Conclusions

This study is particularly relevant to clinical practice, as our cohort was directly recruited from help-seeking outpatients with a wide range of psychiatric comorbidities. Our findings highlight the need for comprehensive assessment in ADHD patients, beyond symptom severity. Treatment should address comorbid personality disorders with targeted approaches like DBT, and particular attention should be given to those with psychiatric hospitalizations. Suicide risk should be assessed broadly, including factors like low self-esteem, not just classic risk profiles. Importantly, the identification of low self-esteem as a factor associated with nonviolent suicide attempts offers a novel and clinically meaningful insight. This finding may enhance suicide risk stratification and inform more tailored preventative interventions within this population.

## Figures and Tables

**Table 1:** Sociodemographic and clinical characteristics of ADHD patients with and without a history of suicide attempts.

	Total Sample (n = 211)	SA Group (21)	Non-SA Group (190)	*p*-Value
Sex, n (%)				0.614
Male	141 (66.8)	13 (61.9)	128 (67.4)	
Female	70 (33.2)	8 (38.1)	62 (32.6)	
Age (years), mean (SD)	27.41 (9.380)	28 (10.085)	27.27 (9.339)	0.735
Age at diagnosis (years), mean (SD)	24.9 (10.336)	24.71 (9.885)	24.90 (10.414)	0.939
Age at first ADHD treatment (years), mean (SD)	25.1 (9.793)	25.24 (9.196)	25.08 (9.876)	0.950
**Educational level, mean (SD)**	12 (3.284)	**11.10 (2.809)**	**13.09 (3.304)**	**0.008**
Current occupation, n (%)				0.109
Unemployed	61 (28.9)	12 (57.1)	49 (25.8)	
Student	56 (26.5)	4 (19.0)	54 (28.4)	
Worker	94 (44.6)	5 (23.9)	87 (45.8)	
Marital status, n (%)				0.144
Single	178 (84.4)	20 (95.2)	159 (83.6)	
Married/cohabiting	28 (13.3)	0 (0)	27 (14.2)	
Separated	5 (2.4)	1 (4.8)	4 (2.1)	
**Current smoking**	**89 (42.2)**	**14 (66.7)**	**74 (38.9)**	**0.016**
**Physical activity**	**96 (45.5)**	**5 (23.8)**	**91 (47.9)**	**0.032**
Family history of psychiatric disorders, n (%)	97 (45.9)	8 (38.1)	89 (46.8)	0.397
**Adult ADHD subtype, n (%)**				**0.026**
**Inattentive subtype**	99 (46.9)	**4 (19.0)**	**95 (50.0)**	
**Combined subtype**	112 (53.1)	**17 (81.0)**	**95 (50.0)**	
Childhood ADHD subtype, n (%)				0.108
Inattentive subtype	75 (35.5)	4 (19.0)	71 (37.4)	
Combined subtype	136 (64.5)	17 (81.0)	119 (62.6)	
BIS-11, mean (SD)	70.4 (11.679)	77.5 (8.668)	69.78 (11.743)	0.77
ADHD-RS pre-treatment, mean (SD)	35.5 (8.267)	35.07 (7.583)	35.56 (8.378)	0.828
Number of symptoms in childhood, mean (SD)				
Inattentive	7.3 (1.316)	7.69 (1.109)	7.35 (1.336)	0.369
Hyperactive–impulsive	5.3 (2.812)	6.46 (2.402)	5.28 (2.844)	0.149
Number of symptoms in adulthood, mean (SD)				
**Inattentive**	7.2 (1.464)	**8.00 (1.155)**	**7.12 (1.473)**	**0.038**
Hyperactive–impulsive	5.3 (2.473)	6.46 (2.402)	5.24 (2.465)	0.115
Lifetime psychiatric comorbidities, n (%)				
**Any comorbid disorder**	152 (72.0)	**21 (100)**	**131 (68.9)**	**0.002**
**Personality disorders**	30 (14.2)	**11 (52.4)**	**19 (10.0)**	**<0.001**
**Substance use disorders**	64 (30.3)	**11 (52.4)**	**53 (27.9)**	**0.016**
**Stimulant use disorder**	37 (17.5)	**7 (33.3)**	**30 (15.8)**	**0.048**
Alcohol use disorder	8 (3.8)	2 (9.5)	6 (3.1)	0.151
Cannabis use disorder	52 (24.6)	8 (38.1)	44 (23.1)	0.123
Major depressive disorder	58 (27.5)	8 (38.1)	50 (26.3)	0.264
Bipolar disorders	23 (10.9)	2 (9.5)	21 (11.0)	0.933
Areas of functional impairment, n (%)				
**Social functioning**	123 (58.3)	**19 (90.5)**	**104 (54.7)**	**0.008**
**Relational functioning**	152 (72.0)	**21 (100)**	**131 (68.9)**	**0.003**
Academic functioning	192 (90.9)	19 (90.5)	173 (91.0)	0.740
Occupational functioning	153 (72.5)	19 (90.5)	134 (70.5)	0.155
Related symptoms, n (%)				
**Mood swings**	155 (73.5)	**20 (95.2)**	**135 (71.0)**	**0.020**
**Anger outbursts**	114 (54.0)	**17 (81.0)**	**97 (51.0)**	**0.010**
Low self-esteem	156 (73.9)	19 (90.5)	137 (72.1)	0.077
**Low tolerance of frustrations**	145 (68.7)	**21 (100)**	**124 (65.3)**	**0.001**
**Number of hospitalizations**	0.6 (3.618)	**4.38 (10.712)**	**0.23 (0.825)**	**<0.001**

Notes: Sociodemographic and clinical characteristics of the total sample (n = 211) are presented with a comparison between patients with (SA group) and without (non-SA group) a history of suicide attempts. Group comparisons were performed using chi-square (χ^2^) tests for categorical variables and the Kruskal–Wallis H test for continuous variables. Statistical significance was set at *p* < 0.05. SA = suicide attempt; SD = standard deviation. BIS-11 = Barratt Impulsiveness Scale-11, a self-report questionnaire assessing cognitive, motor, and non-planning impulsivity; ADHD-RS = Attention Deficit Hyperactivity Disorder Rating Scale-IV, an 18-item self-report questionnaire designed to assess ADHD symptoms based on DSM-IV diagnostic criteria.

**Table 2:** Sociodemographic and clinical characteristics of ADHD patients with violent and nonviolent suicide attempts.

	Violent SA (5)	Nonviolent SA (16)	*p*-Value
Sex, n (%)			0.340
Male	4 (80.0)	9 (56.2)	
Female	1 (20.0)	7 (43.8)	
Age (years), mean (SD)	28.60 (10.237)	27.81 (10.368)	0.883
Age at diagnosis (years), mean (SD)	23.80 (14.653)	25 (8.524)	0.820
Age at first ADHD treatment (years), mean (SD)	28.49 (10.644)	23.92 (8.681)	0.377
Educational level	11.60 (3.507)	10.94 (2.670)	0.657
Current occupation, n (%)			0.742
Unemployed	2 (40.0)	10 (62.5)	
Student	1 (20.0)	3 (18.8)	
Worker	2 (40.0)	3 (18.8)	
Marital status, n (%)			0.067
Single	4 (80.0)	16 (100)	
Married/cohabiting	0 (0.0)	0 (0.0)	
Separated	1 (20.0)	0 (0.0)	
Current smoking	3 (60.0)	11 (68.8)	0.717
Physical activity	2 (40.0)	3 (18.8)	0.330
Family history of psychiatric disorders, n (%)	4 (80.0)	9 (56.3)	0.340
Adult ADHD subtype, n (%)			0.214
Inattentive subtype	0 (0)	4 (25.0)	
Combined subtype	5 (100)	12 (75.0)	
Childhood ADHD subtype, n (%)			0.214
Inattentive subtype	0 (0)	4 (25.0)	
Combined subtype	5 (100)	12 (75.0)	
BIS-11, mean (SD)	85.50 (13.435)	74.83 (5.913)	0.141
ADHD-RS pre-treatment, mean (SD)	37.67 (12.741)	34.42 (6.431)	0.527
Number of symptoms in childhood, mean (SD)			
Inattentive	8.67 (0.577)	7.40 (1.075)	0.081
Hyperactive–impulsive	7.67 (1.528)	6.10 (2.558)	0.343
Number of symptoms in adulthood, mean (SD)			
Inattentive	8.00 (1.000)	8.00 (1.247)	1.000
Hyperactive–impulsive	7.67 (1.528)	6.00 (2.667)	0.333
Lifetime psychiatric comorbidities, n (%)			
Any psychiatric comorbidities	5 (100)	16 (100)	—
Personality disorders	2 (40.0)	9 (56.3)	0.525
Substance use disorders	2 (40.0)	9 (56.3)	0.525
Stimulant use disorder	2 (40.0)	5 (31.3)	0.717
Alcohol use disorder	1 (20.0)	1 (6.3)	0.361
Cannabis use disorder	1 (20.0)	7 (43.8)	0.340
Major depressive disorder	2 (40.0)	6 (37.5)	0.920
Bipolar disorders	0 (0.00)	2 (12.5)	0.406
Areas of functional impairment, n (%)			
Social functioning	5 (100)	14 (87.5)	0.406
Relational functioning	5 (100)	16 (100)	—
Academic functioning	4 (80.0)	15 (93.8)	0.361
Occupational functioning	4 (80.0)	15 (93.8)	0.166
Related symptoms, n (%)			
Mood swings	5 (100)	15 (93.8)	0.567
Anger outbursts	4 (80.0)	13 (81.3)	0.950
**Low self-esteem**	**3 (60.0)**	**16 (100)**	**0.008**
Low tolerance of frustrations	5 (100)	16 (100)	—
Number of psychiatric hospitalizations	1.20 (1.095)	5.38 (12.176)	0.461

Notes: Sociodemographic and clinical characteristics of the total sample (n = 211) are presented with a comparison between patients with violent (violent SA group) and nonviolent (nonviolent SA group) suicide attempts. Group comparisons were performed using chi-square (χ^2^) tests for categorical variables and the Kruskal–Wallis H test for continuous variables. Statistical significance was set at *p* < 0.05. SA = suicide attempt; SD = standard deviation.

**Table 3:** Relationship between potential explanatory variables and lifetime suicide attempts.

	B	SE	Wald	*p*-Value	OR	95% CI
Educational level	−0.010	0.104	0.010	0.920	0.990	0.801–1.212
Current smoking	−0.651	0.847	0.590	0.553	0.522	0.103–2.918185–1.676
Physical activity	−1.127	0.684	2.719	0.099	0.324	0.083–1.204
Adult ADHD subtype	16.574	2559.3	0.000	0.759	5771.7	0.425–9–275
No. of inattentive symptoms in adult	0.721	0.518	1.937	0.164	2.057	0.761–2.465
Lifetime psychiatric comorbidities						
Any comorbid disorder	16.640	4197.9	**0.000**	0.997	14,579.3	0.000
**Personality disorders**	**1.889**	**0.688**	**7.536**	**0.006**	**6.613**	**1.729–20.847**
Substance use disorders	−1.452	1.083	**1.797**	0.180	0.180	**0.023–1.632**
Stimulant use disorder	1.065	0.978	**1.185**	0.276	0.276	0.526–9.432
Mood swings	0.777	1.236	0.395	0.425	2.176	0.235–31.052
Anger outbursts	−0.016	0.861	0.000	0.985	0.984	0.179–5.650
Low tolerance of frustrations	21.314	2577.5	0.000	0.997	37,945.6	0.000
Social functioning impairment	−0.239	0.626	0.146	0.659	0.788	0.128–3.678
Relational functioning impairment	24.997	6510.5	0.000	0.997	71,765	0.000
**Number of psychiatric hospitalizations**	**0.683**	**0.256**	**7.124**	**0.008**	**1.980**	**0.296–2.675**
Constant	−65.176	7559.5	0.000	0.993	0.000	−88.49–−51.85

Notes: Results from the binary logistic regression analysis examining the relationship between potential explanatory variables and lifetime suicide attempts in patients with ADHD (n = 211) are presented. Statistical significance was set at *p* < 0.05.

## Data Availability

The raw data supporting the conclusions of this article will be made available by the authors on request.
